# Supplementary material for: Molecular Dynamics Study on Behavior of Resist Molecules in UV-Nanoimprint Lithography Filling Process
Source: Nanomaterials (Basel). 2022 Jul 25;12(15):2554. doi: 10.3390/nano12152554 (PMC9331815; doi:10.3390/nano12152554)
Supplement: Supplementary file 1 [file nanomaterials-12-02554-s001.zip › nanomaterials-1752434-supplementary.pdf]

nanomaterials

Supplementary Materials

Molecular Dynamics Study on Behavior of Resist Molecules in UV-Nanoimprint Lithography Filling Process

Jun Iwata and Tadashi Ando *

Department of Applied Electronics, Faculty of Advanced Engineering, Tokyo University of Science, Tokyo 125-8585, Japan; 8121503@ed.tus.ac.jp
* Correspondence: tando@rs.tus.ac.jp

Citation: Iwata, J.; Ando, T. Molecular Dynamics Study on Behavior of Resist Molecules in UV-Nanoimprint Lithography Filling Process. *Nanomaterials* **2022**, *12*, 2554. https://doi.org/10.3390/nano12152554

Academic Editor: Gregory M. Odegard

Received: 17 May 2022
Accepted: 21 July 2022
Published: 25 July 2022

Publisher's Note: MDPI stays neutral with regard to jurisdictional claims in published maps and institutional affiliations.

Copyright: © 2022 by the authors. Submitted for possible open access publication under the terms and conditions of the Creative Commons Attribution (CC BY) license (http://creativecommons.org/licenses/by/4.0/).

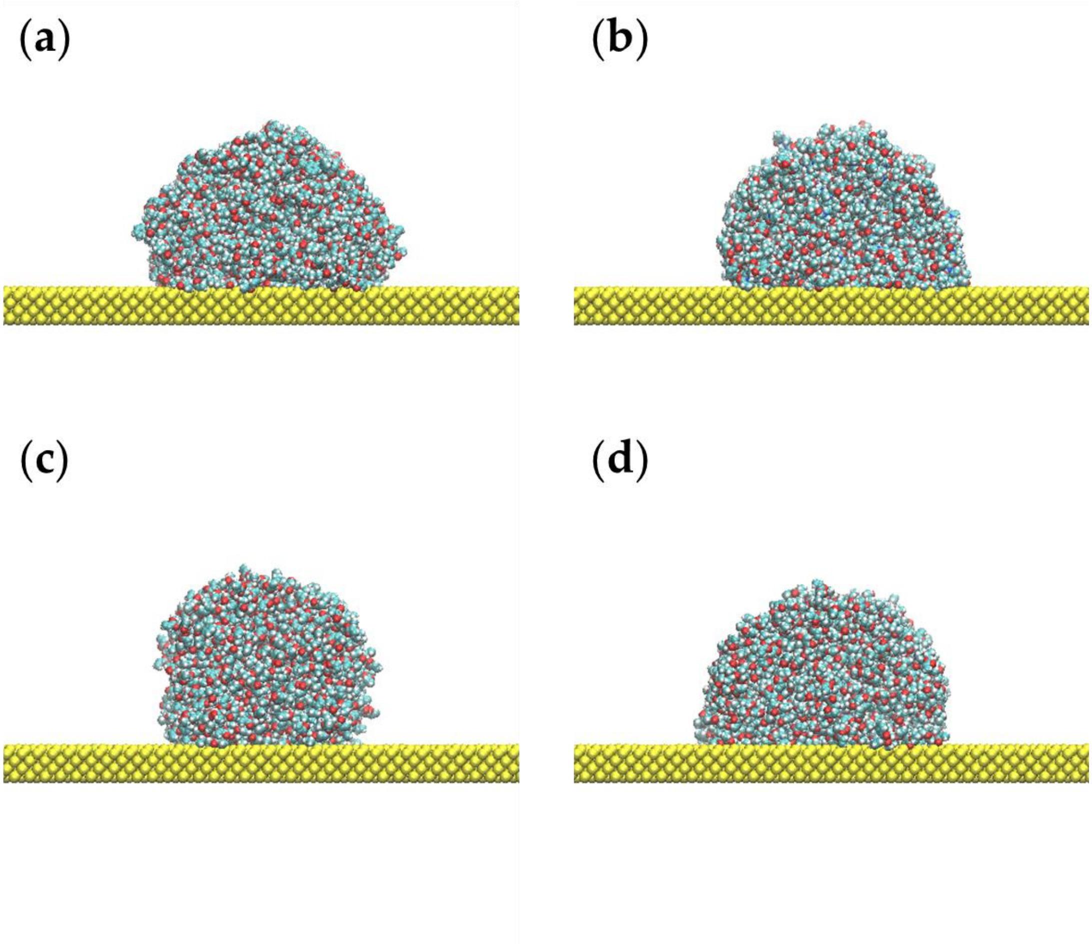

Figure S1. Snapshots of the systems for contact angle estimation at the end of the simulations for four resists: (**a**) resist I at 200 ns; (**b**) resist II at 200 ns; (**c**) resist III at 650 ns; and (**d**) resist IV at 500 ns.

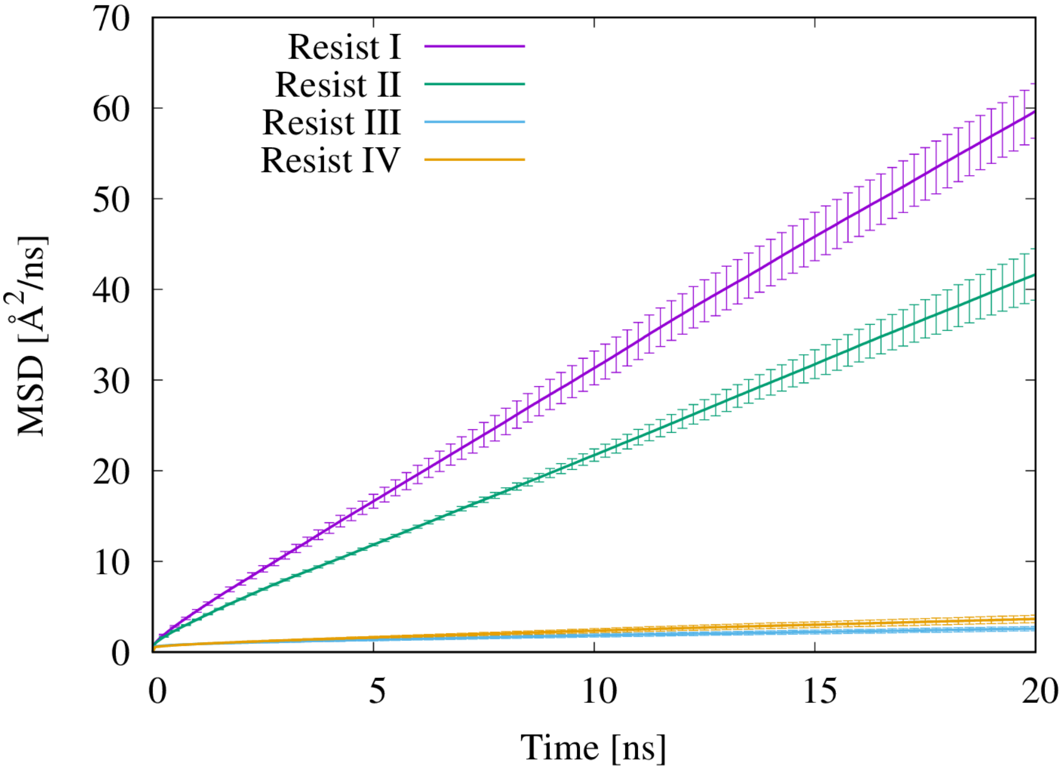

Figure S2. Mean square displacement (MSD) of DMPA molecules in the four resists as a function of time. MSD curves are averaged over the DMPA molecules in the simulation box and five independent simulations. Error bars are the standard error calculated from the five independent simulations. The diffusion coefficients were evaluated from the slope of the MSD between 5 and 20 ns.

Table S1. Weight ratios of the molecules filling the trenches with Δ of 3 and 2 nm in resist II system.[1]

**Molecule**	**Δ = 3 nm**	**Δ = 2 nm**	**Bulk**
TMPTA	10.0	10.0	10.0
TPGDA	55.5	56.4	57.0
NVP	31.2	30.8	29.0
DMPA	3.3	2.8	4.0

[1] All values represent weight percentages.

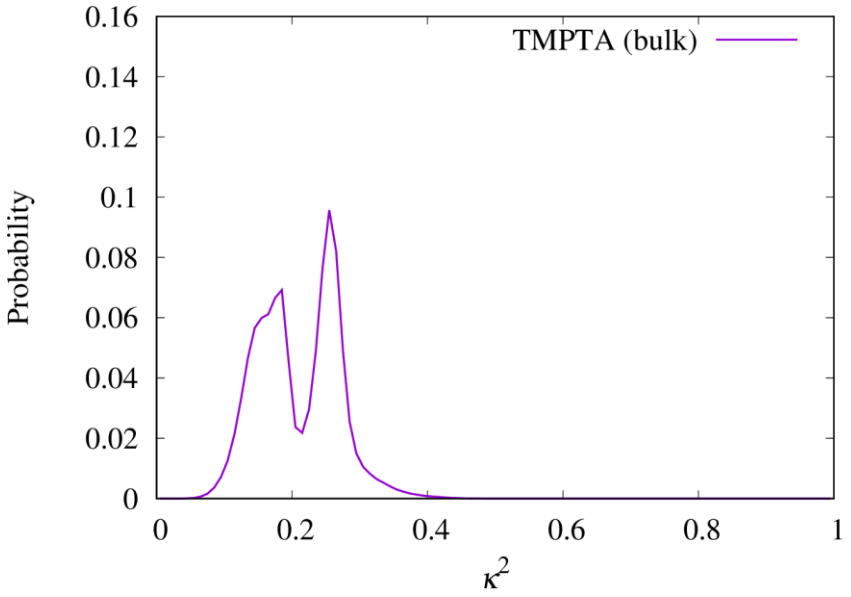

Figure S3. Distributions of the relative shape anisotropy, κ^2, of TMPTA in the bulk state of resist III.

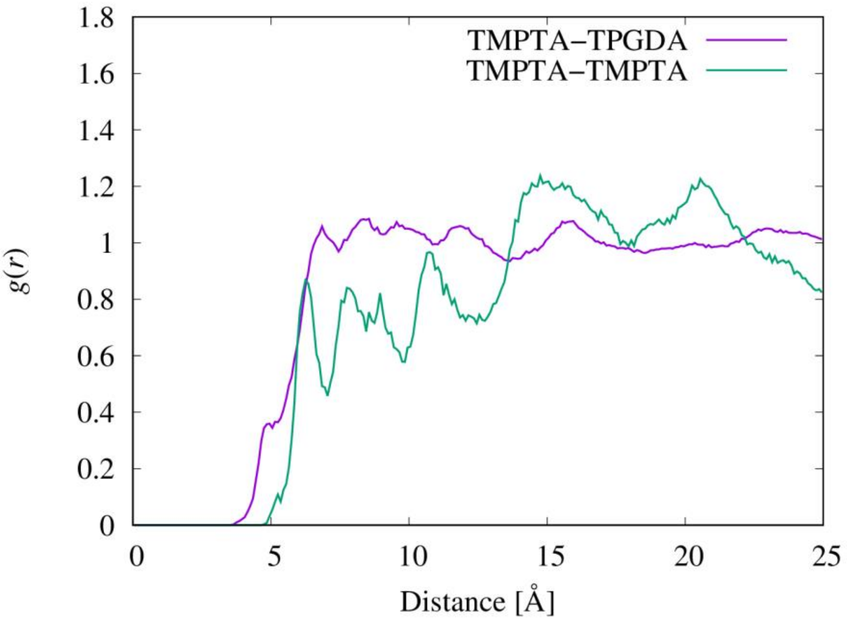

Figure S4. Radial distribution function, $g(r)$, between the centers of masses of the TMPTA–TPGDA and TMPTA–TMPTA pairs in resist II.
